# Paclitaxel Induces Apoptosis in AIDS-Related Kaposi's Sarcoma Cells

**DOI:** 10.1155/S1357714X00000074

**Published:** 2000-03

**Authors:** Jie Cai, Tong Zheng, Rizwan Masood, D. Lynne Smith, David R. Hinton, Caryn Nae Kim, Guofu Fang, Kapil Bhalla, Parkash S. Gill

**Affiliations:** 1 Departments of Medicine University of Southern California Kenneth Norris Comprehensive Cancer Center and Research Institute Los Angeles CA USA; 2 Departments of Pathology University of Southern California Kenneth Norris Comprehensive Cancer Center and Research Institute Los Angeles CA USA; 3 Division of Clinical and Translational Research Sylvester Comprehensive Cancer Center University of Miami FL USA

## Abstract

Paclitaxel is a microtubule stabilizing drug that causes dividing cells to arrest and then undergo apoptosis. It also has antiangiogenic activity because it alters cytoskeletal structure, affecting migration and invasion. Paclitaxel is an effective treatment for AIDS-related Kaposi’s sarcoma (KS). KS is a tumor in which there is marked proliferation of endothelial cells in addition to the tumor cells, which themselves share many markers with activated (proliferating) endothelial cells.We sought to determine the mechanism by which paclitaxel exerts its anti-KS tumor effects. *In vitro*, KS cells are very sensitive to paclitaxel, with half-maximal growth inhibition observed at 0.8 nM. Inhibition of migration of KS cells was also observed at nanomolar concentrations of the drug. Paclitaxel induced cell cycle arrest with an accumulation of cells in sub-G1.This was accompanied *in vitro* by various events typical of apoptosis: phosphorylation of two anti-apoptotic proteins Bcl-2 and Bcl-_xL_ , release of cytochrome c into the cytoplasm, cleavage and activation of caspase-3. *In vitro* results were borne out by studies of KS tumor xenografts in nude mice. Paclitaxel (10 mg/kg) inhibited tumor growth by 75% over 21 days. Histological examination of the tumors revealed a decrease in proliferative index, a decrease in the number of mitotic figures and an increase in apoptotic cells compared to tumors from untreated mice.


Sarcoma (2000) 4, 37–45

ORIGINAL ARTICLE

### Paclitaxel induces apoptosis in AIDS-related Kaposi's sarcoma cells

JIE CAI,[1] TONG ZHENG,[1] RIZWAN MASOOD,[2] D. LYNNE SMITH,[1] DAVID R. HINTON,[2] CARYN NAE KIM,[3] GUOFU FANG,[3] KAPIL BHALLA[3] & PARKASH S. GILL[1]

[1]*Departments of Medicine and* [2]*Pathology, University of Southern California, Kenneth Norris Comprehensive Cancer Center and Research Institute, Los Angeles, CA, USA,* [3]*Division of Clinical and Translational Research, Sylvester Comprehensive Cancer Center, University of Miami, FL, USA*

**Abstract**

Paclitaxel is a microtubule stabilizing drug that causes dividing cells to arrest and then undergo apoptosis. It also has anti-angiogenic activity because it alters cytoskeletal structure, affecting migration and invasion. Paclitaxel is an effective treatment for AIDS-related Kaposi's sarcoma (KS). KS is a tumor in which there is marked proliferation of endothelial cells in addition to the tumor cells, which themselves share many markers with activated (proliferating) endothelial cells. We sought to determine the mechanism by which paclitaxel exerts its anti-KS tumor effects. *In vitro*, KS cells are very sensitive to paclitaxel, with half-maximal growth inhibition observed at 0.8 nM. Inhibition of migration of KS cells was also observed at nanomolar concentrations of the drug. Paclitaxel induced cell cycle arrest with an accumulation of cells in sub-G1. This was accompanied *in vitro* by various events typical of apoptosis: phosphorylation of two anti-apoptotic proteins Bcl-2 and Bcl-$x_L$, release of cytochrome c into the cytoplasm, cleavage and activation of caspase-3. *In vitro* results were borne out by studies of KS tumor xenografts in nude mice. Paclitaxel (10 mg/kg) inhibited tumor growth by 75% over 21 days. Histological examination of the tumors revealed a decrease in proliferative index, a decrease in the number of mitotic figures and an increase in apoptotic cells compared to tumors from untreated mice.

**Key words**: *AIDS, apoptosis, Kaposi's sarcoma, paclitaxel, Taxol*

### Introduction

Kaposi's sarcoma is the most common tumor in patients with human immunodeficiency virus type 1 (HIV-1) infection.[1] Clinically, KS manifests most commonly on the skin and mucus membranes as multicentric lesions, with subsequent spread to visceral organs.[1] KS-related mortality is secondary to visceral organ disease. There has been debate as to whether KS is a true neoplasm, or merely represents a local hyperproliferation reaction in response to inflammatory cytokines and growth factors. With the results of two groups that show clonality in KS lesions this debate appears to be resolving in favor of the neoplastic nature of the disease.[2,3]

KSHV/HHV-8, a recently identified herpes virus, is a strong candidate for the etiologic agent for all forms of KS, whether classical, AIDS-associated or endemic. Molecular epidemiology studies have shown that nearly all KS tumors contain viral genome, suggesting an etiological link to this virus.[4] Sero-epidemiology studies show that KS patients have a very high prevalence of KSHV/HHV8 antibodies (90%) which is elevated above the non-KS population (5–20% in the USA). Further, in countries with HIV but no documented KS, KSHV seroprevalence is very low (2–4%).[5]

Pathologically, the tumor exhibits an extensive vascular network of slit-like spaces, which differ from normal vessels in that they lack basement membranes and the presence of abnormal spindle-shaped endothelial cells (tumor cells) lining these vessels. The pathological examination of the tumors suggests that the origin of KS tumor cells is endothelial, a hypothesis that is supported by phenotypic studies. Markers shared with endothelial cells include lectin binding sites for *Ulex europeaus* agglutinin-1 (UEA-1), CD34, EN-4, PAL-E,[6] ELAM-1,[7] VE-cadherin[8] and the recently identified endothelial cell specific tyrosine kinase receptors, VEGFR-1 (Flt-1), VEGFR-2 (Flk-1/KDR), VEGFR-3 (Flt-4), Tie-1 and Tie-2.[9–11] In addition to expressing many endothelial proteins, KS cell growth is strongly dependent on the angiogenic factors vascular endothelial growth factor (VEGF),[9] basic fibroblast growth factor (bFGF),[12]

Correspondence to: Parkash S. Gill, MD, Professor of Medicine and Pathology, Kenneth Norris Jr. Cancer Hospital and Research Institute, Room 3458, 1441 Eastlake Avenue, Los Angeles, CA 90033, USA. Tel: (+1) 323-865-3909; Fax: (+1) 323-865-0060; E-mail: parkashg@hsc.usc.edu

1357-714X print/1369-1643 online/00/010037-09 © 2000 Taylor & Francis Ltd and to a lesser extent, interleukin-8 (R.M., T.Z., P.S.G., unpublished data).

Paclitaxel has shown potent anti-tumor activity in several tumor types.[13–16] Paclitaxel increases tubulin polymerization, which results in cell-cycle arrest due to inhibition of mitotic spindle formation.[17] Prolonged cell-cycle arrest has been associated with phosphorylation of the Bcl-2 protein.[18] The Bcl-2 family of proteins plays a central role in programmed cell death or apoptosis, in which anti-apoptotic members such as Bcl-2 and Bcl-$x_L$ balance the activities of the pro-apoptotic members such as Bax and Bad.[19,20] Heterodimerization of Bcl-2 with Bax is critical in preventing Bax-mediated apoptosis.[21,22] Bcl-2 and Bax have additional opposing actions in apoptosis since Bcl-2 prevents the release of cytochrome c from mitochondria[23] while Bax promotes cytochrome c release.[24] Cytochrome c release results in binding to Apaf-1 followed by activation of caspase 9 and effector caspases[25] that are active in the effector phase of apoptosis.[26]

In addition to inducing apoptosis, paclitaxel may also exert its anti-tumor activity through inhibition of angiogenesis. Paclitaxel alters cytoskeletal structures and inhibits endothelial cell migration *in vitro* and *in vivo*.[27] Paclitaxel has also been shown to inhibit angiogenesis in the chicken chorio-allantoic membrane (CAM) assay[28] and bFGF induced angiogenesis in the mouse corneal neovascularization model.[29]

Since KS is a highly vascular tumor we first sought to determine the sensitivity of KS to paclitaxel *in vitro* and *in vivo*. We found that very low (nanomole) concentrations of paclitaxel induced tumor cell death. We show that the mechanism of anti-tumor action of paclitaxel in KS is accompanied by phosphorylation of Bcl-2, release of cytochrome c into the cytoplasm and activation of caspase-3.

### Materials and methods

### *Cell proliferation assays*

The immortalized KS cell lines KS Y-1[30] and KS-SLK[31] were grown in wells coated with 1.5% gelatin in KS medium consisting of RPMI-1640 (Life Technologies, Gaithersburg, MD), 100 U/ml penicillin, 100 μg/ml streptomycin, 2 mM glutamine, 1% essential and non-essential amino acids, 10% fetal bovine serum (FBS: Life Technologies), and 1% Nutridoma-HU (Boehringer Mannheim, Indianapolis, IN). HUVEC (Clonetics, San Diego, CA) were grown in medium containing epidermal growth factor and according to the instructions of the supplier. Cells were plated at a density of 10 000 cells/well in 48-well gelatin coated plates on day 0. Similarly, AoSM (Clonetics, San Diego) cells were seeded in 48 well plates at the same density in their growth media on day 0. On days 1 and 3 cells were treated with various concentration of paclitaxel. On day 5, cells were treated with 3-[4,5-dimethylthiazol-2-yl]-2,5-diphenyltetrazolium bromide (MTT) at a final concentration of 0.5 mg/ml. Cells were incubated for 2 hours, medium was aspirated and the cells were dissolved in acidic isopropanol (90% isopropanol, 0.5% SDS and 40 mM HCl). Developed color was read in an ELISA reader using the isopropanol as blank (Molecular Devices, CA).

### *Cell migration assays*

Cell migration assays were performed in transwells with 8 μm pores (Costar, Cambridge, MA). Briefly, wells were coated with fibronectin (25 μg/ml) overnight, and the endothelial cells or KS cells were plated in 100 μl of DMEM/0.4% FCS in the upper chamber. 500 μl of DMEM/0.4% FCS was added to the lower chamber and incubated at 37°C for 1 hour. Paclitaxel (0.01–100 nM) was added to the upper chamber, and chemotaxis agents (VEGF 20 ng/ml) to the lower chamber. The plates were incubated for 16 hours at 37°C and the cells crossing the fibronectin-plated membrane were quantitated after wiping the cells off the upper chamber with a cotton swab. The cells across the membrane were stained with Diff-Quik stain set according to the manufacturer's instruction (Dade Diagnostics Inc., Aguada, PR). The cells were counted at 320 X in four randomly selected fields. The experiments were done in duplicate and repeated at least three times.

### *Apoptosis assays*

Internuclear fragmentation of genomic DNA was assessed after incubation of the cells with paclitaxel. DNA from 1μ10[6] cells was extracted and size-fractionated by agarose gel electrophoresis as described previously.[32] KS cells were also analyzed by flow cytometry for cell cycle and apoptosis after incubation with paclitaxel. The cell pellets were fixed in 70% alcohol, washed and the nuclei were stained with propidium iodide solution for analysis as described previously.[33] The samples were read on a Coulter Elite flow cytometer using Elite software program 4.0 for two-color detection. The percentage of cells in the apoptotic sub-G1, G1, S, and G2-M phase were calculated using Multicycle software (Phoenix Flow Systems, San Diego, CA).

### *Protein analysis*

The levels of Bcl-$x_L$, Bcl-2 and Bax and their phosphorylation status, levels of cytosolic cytochrome c, activation of caspase-3, proteolysis of poly (ADP-ribose) polymerase (PARP), and cleavage product of DNA fragment factor (DFF) in control cells and in response to paclitaxel treatment were also examined in KS cells. These assays were performed by Western blot using specific antibodies conjugated to horseradish peroxidase.[34–38]

### In vivo *tumor growth*

*In vivo* studies were performed in BALB/c $NU^+/nu^+$ athymic mice by implanting KS Y-1 cells (10 million cells each) subcutaneously. Mice were treated with paclitaxel (5 and 10 mg/kg) or an equal amount of vehicle control intraperitoneally on days 1, 5, 9, 14 and 21. Tumor sizes were measured on days 14 and 21. Tumor volume was calculated with the formula $V = 0.52LW^2$. Six mice were treated in each group. Mice were handled throughout in compliance with IACUC regulations.

### *Histological analysis of* in vivo *tumors*

In other experiments, tumors were allowed to grow for 14 days and treated with 5, 10, or 20 mg/kg of paclitaxel. Tumors were excised 24 hours later, fixed in 4% paraformaldehyde, imbedded in paraffin, sectioned and analyzed for the mitotic index, and apoptosis *in situ*. Immunoperoxidase staining for the proliferation-related antigen, Ki-67 was performed using MIB-1 monoclonal antibody (Immunotech, Marseilles, France). After incubation with the primary antibody, staining was completed using the ABC immunoperoxidase method on an automated stainer (Techmate 1000, BioTek, Santa Barbara, CA). Diaminobenzidine (DAB) was used as the peroxidase substrate. TUNEL staining for apoptosis was performed using the ApopTag *in situ* apoptosis detection kit (Oncor, Gaithersburg, MD) according to the protocol supplied by the manufacturer. In this method cut DNA is localized *in situ* by end-labeling, using terminal deoxyribonucleotidyl transferase (TdT) and an digoxigenin-deoxyuridine5-triphosphate substrate. The bound digoxigenin is detected immunohistochemically using antidigoxigenin–peroxidase antibodies. Peroxidase was visualized using aminoethylcarbizole (AEC) as the chromogen (AEC kit, Zymed Lab Inc., San Francisco, CA). Negative controls included substitution of water for TdT. For each tumor, cell density was determined on H&E sections by counting the number of nuclei in 5 representative 1 $mm^2$ sections. The number of morphologically identifiable mitotic figures was counted in these same regions of the H&E stained sections. The labeling index for MIB-1 and TUNEL positive cells was performed by determining the percentage of labeled nuclei in 5 representative 1 $mm^2$ sections from each tumor.

### Results

### *Paclitaxel inhibits KS cell proliferation and migration*

We examined the effects of paclitaxel on KS cell proliferation *in vitro*. The 50% inhibitory concentration ($IC_{50}$) for paclitaxel was 0.8 nM, and 90% inhibition was observed at 10 nM (Fig. 1a). In contrast, endothelial cells (HUVEC) and aortic smooth muscle cells (AoSM), were less sensitive, with $IC_{50}$ of 6.6 nM and 4.7 nM, respectively. We also investigated the effects of paclitaxel on KS cell migration in response to bFGF. Paclitaxel inhibited KS cell migration with an $IC_{50}$ of 1.1 nM (Fig. 1b). Inhibition of migration (16 hours) occurred earlier than inhibition of proliferation (5 days). For all concentrations of paclitaxel except the highest tested (100 nM), we do not observe cell death at 16 hours.

### *Paclitaxel induces apoptosis in KS cells*

Exposure of KS cells to paclitaxel produced a dose-dependent increase in internucleosomal DNA fragmentation (Fig. 2a). This was associated with an increase in the fraction of sub-G1 cells with DNA content below that of the interphase resting cells (<2N) (Fig. 2b). Annexin V is translocated to the cell

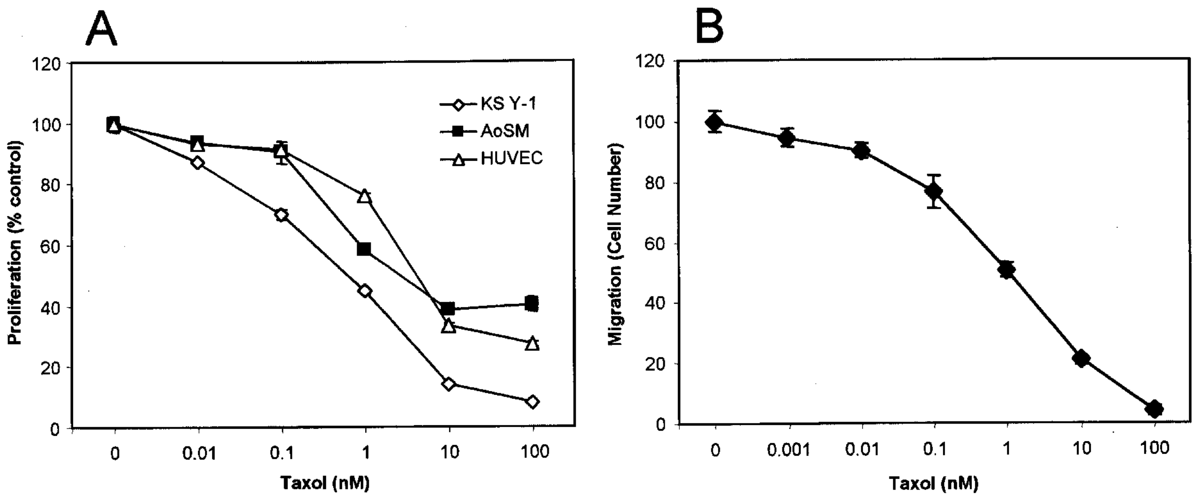

**Figure 1.** *Taxol inhibits proliferation and migration of KS cells. (a) KS Y-1, HUVEC and AoSM cells were plated at equal density (1μ $10^3$ cells/well) and treated with Taxol on days 1 and 3 at the concentrations shown. Cells were counted on day 5. (b) migration assays were done in double chamber wells separated by a fibronectin-coated membrane. Chemotaxis was induced by addition VEGF (20 ng/ml) to the lower chamber. Cells (5μ $10^4$/ml) were placed in the upper chamber in the presence and absence of paclitaxel at the concentration shown. Migration of KS Y-1 cells across the membrane was quantitated after overnight incubation. The results represent the mean ± standard error of assays performed in quadruplicate.*

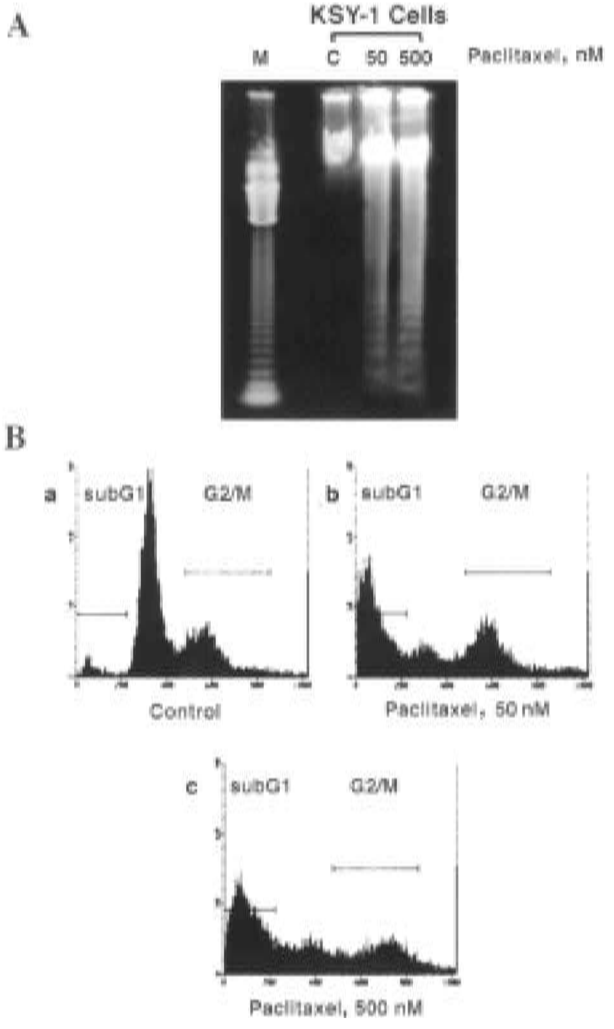

**Figure 2.** *Taxol induces internucleosomal DNA fragmentation and increases the fraction of sub-G1 cells with <2N DNA content. (a) Following exposure of KS Y-1 cells to 50 or 500 nM Taxol for 24 hours, the genomic DNA was extracted, purified and subjected to agarose gel electrophoresis. (b) Flow cytometric determination of DNA content and cell cycle status of KS Y-1 cells treated with Taxol for 24 hours compared with untreated cells (control). The areas of the plots that represent cells in sub-G1 and G2/M phase of the cell cycle are indicated. There is a dose-dependent increase in sub-G1 cells with <2N DNA content.*

surface during apoptosis and is used as a marker of apoptotic cells. We stained KS cells exposed to 0.1 to 100 nM paclitaxel with Annexin V to determine the fraction of apoptotic cells *in vitro*. It can be seen in Fig. 3 that 25% of cells were apoptotic following 24 hour exposure to 100 nM paclitaxel. At 500 nM paclitaxel, the highest concentration used in other experiments reported in this study, 48% of cells exhibited morphologic features of apoptosis.

### *Caspase-3 is activated upon paclitaxel treatment of KS cells*

We further investigated the mechanism of KS cell death in response to paclitaxel. One of the pivotal proteases active in apoptosis is caspase-3, which degrades several protein substrates including poly (ADP-ribose) polymerase (PARP).[37,38] Twenty-four

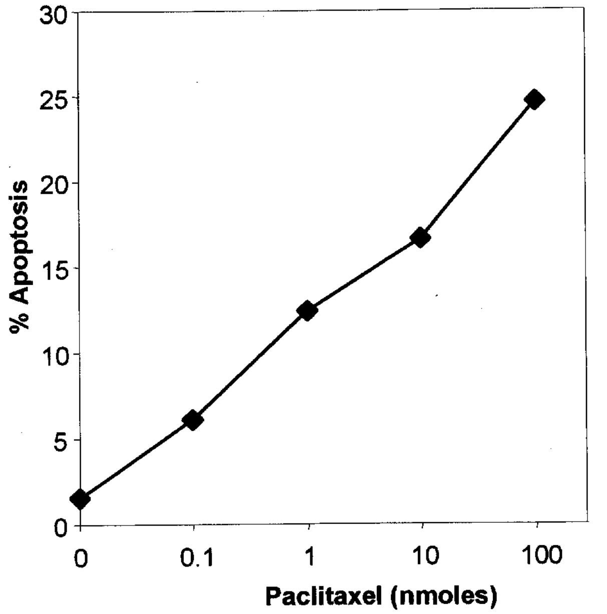

**Figure 3.** *Taxol treatment leads to apoptosis of KS cells. Following exposure of the Kaposi's sarcoma cell line, KS Y-1, to paclitaxel (0.1, 1, 10, and 100 nmol) for 24 hours, the cells were trypsinized and stained for annexin V. Cells displaying annexin V on the cell surface were quantitated by flow cytometry.*

hour treatment of KS cells with paclitaxel resulted in a dose-dependent cleavage of full-length procaspase-3 (Fig. 4a). This was associated with the degradation of caspase-3 substrate PARP into its 85 kD (Fig. 4a) and 31 kD (not shown) fragments.

### *Paclitaxel induces phosphorylation of Bcl-2 and Bcl-$x_L$ in KS cells*

The mechanism of paclitaxel-induced apoptosis involves modulation of regulatory proteins such as Bcl-2, Bcl-$x_L$, and Bax. Figure 4b demonstrates that exposure of KS cells to paclitaxel for 24 hours results in the appearance of slower-mobility species characteristic of the phosphorylated forms of Bcl-2 and Bcl-$x_L$. Under the same treatment Bax levels were unchanged. A time course of KS cell exposure to 500 nM paclitaxel showed that phosphorylation of Bcl-2 and Bcl-$x_L$ had reached a peak by 5 hours, whereas caspase-3 activation measured by cleavage of PARP was not appreciable until 12 hours (Fig. 4c).

### *Cytochrome c is released by paclitaxel treatment in KS cells*

Release of cytochrome c from the mitochondria into the cytosol may precede apoptosis. We determined whether this occurred in KS cells in response to paclitaxel. Paclitaxel, but not etoposide, resulted in cytochrome c release into the cytoplasm after 24 hour treatment (Fig. 5). This release of cytochrome c was associated with the induction of downstream apoptotic events in the same cells. Two substrates of caspase-3, PARP and DFF, were cleaved into smaller forms (Fig. 5).

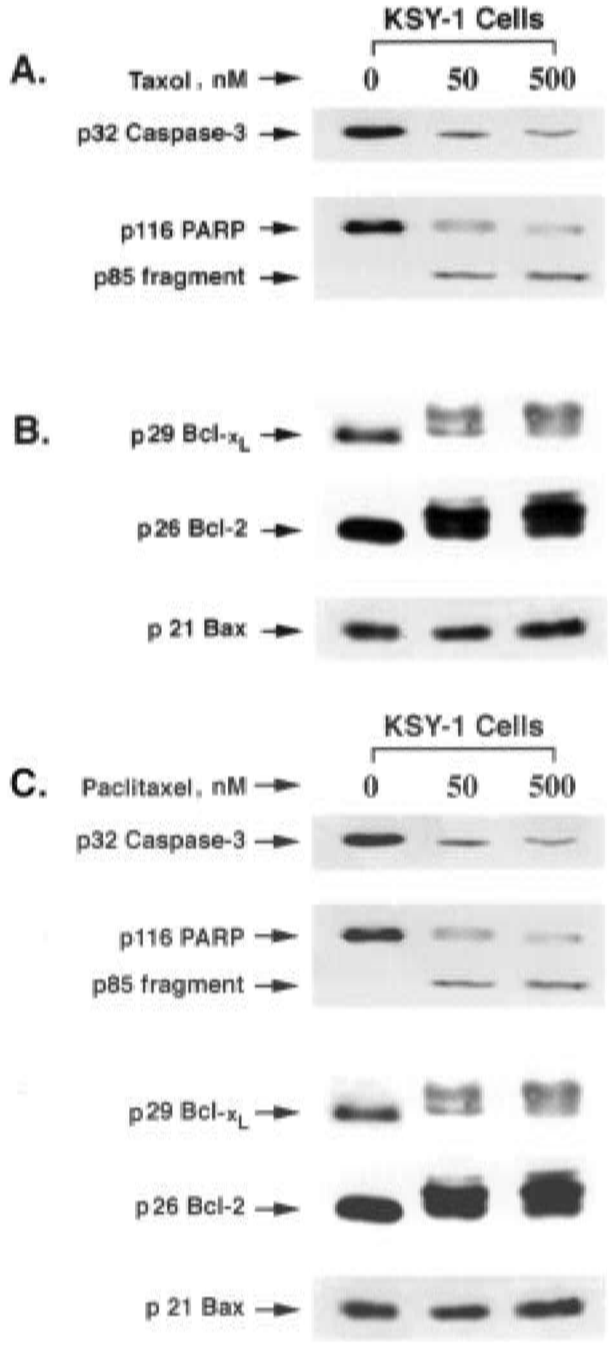

**Figure 4**. *Taxol induces Bcl-2 phosphorylation and the molecular cascade of apoptosis in KS Y-1 cells. KS Y-1 cells were exposed to the indicated concentration of Taxol for 24 hours. Western blot analysis of several apoptotic markers. (a) Decrease in procaspase-3 (p32 caspase-3; by cleavage; see text) and degradation of caspase-3 substrate 116 kD PARP into its 85 kD and 31 kD (not shown) fragments. (b) Treatment with Taxol produced a dose-dependent increase in slower mobility phosphorylated Bcl-2 and Bcl-$x_L$ bands, whereas Bax levels remain unaltered. (c) Western analyses of the levels of p29Bcl-$x_L$, p26Bcl-2, full-length caspase-3, as well as the analysis of the caspase-3 activity by determining the in vitro cleavage of $^{35}$S-labeled PARP by cellular protein extracts from untreated and paclitaxel (500 nM) treated cells for 5, 8, 12, and 24 hours. Exposure to 500 nM paclitaxel for ≥5 hours resulted in a time-dependent increase in the phosphorylation of Bcl-2 and Bcl-$x_L$. A decline in the p32 caspase-3 levels and PARP cleavage activity occurred after exposure to paclitaxel for at least 12 hours.*

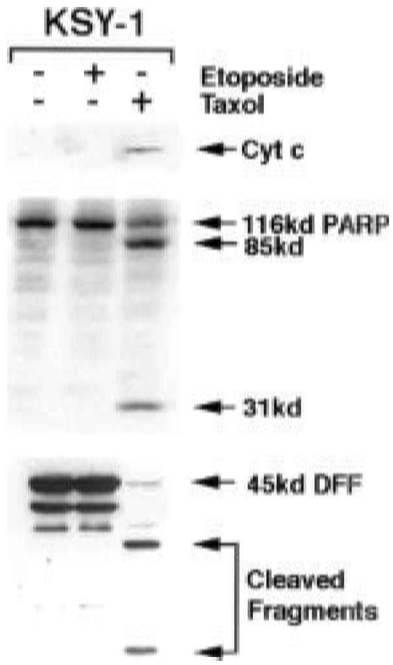

**Figure 5**. *Taxol induces apoptosis in KS Y-1 cells by release of cytochrome c. Western analysis of cytosolic cytochrome c (cyt c) levels and the 45 kD subunit of DNA fragmentation factor (DFF). $^{35}$S-labeled in vitro translated Poly (ADP-ribose) polymerase (PARP) or its 85 and 31 kD cleavage products were analyzed from the S-100 fraction from the untreated, etoposide-treated (50 μmol for 4 hours), or paclitaxel-treated (50 μmol for 24 hours) KS Y-1 cells. Treatment with paclitaxel but not etoposide induced cytosolic accumulation of cytochrome c as well as PARP and DFF-45 cleavage activity.*

### In vivo *effects of paclitaxel on KS tumors*

The effects of paclitaxel on KS tumors *in vivo* were next determined using tumor xenografts in nude mice. Tumor response was observed in mice treated with a paclitaxel dose of 10 mg/kg (Fig. 6a). In other experiments, tumors were excised 24 hours after a single dose of either 5 or 10 mg/kg and studied for apoptosis *in situ*, and for their effect on the mitotic index. A dose-dependent increase in the cells undergoing apoptosis (Fig. 6b) and a decrease in the mitotic index were observed (Fig. 6c). Comparison of the histology of the control and paclitaxel treated tumors revealed frequent mitotic figures in the control tumors (Fig. 6d) which were reduced upon paclitaxel treatment (Fig. 6e). Immunoperoxidase staining for the proliferation marker Ki-67 showed a significant reduction in paclitaxel treated compared to control tumor (Figs 6g, f). TUNEL staining was performed to quantify the *in vivo* apoptosis. When compared to control (Fig. 6h) paclitaxel treated tumor (Fig. 6i) showed a marked increase in the number of TUNEL-positive cells. When adjusted for cell density, the increase in apoptosis was even more prominent.

### Discussion

Paclitaxel has proven effective in the treatment of a variety of cancers[13–15] including Phase II trials in Kaposi's sarcoma.[16,39] Because of its highly vascular

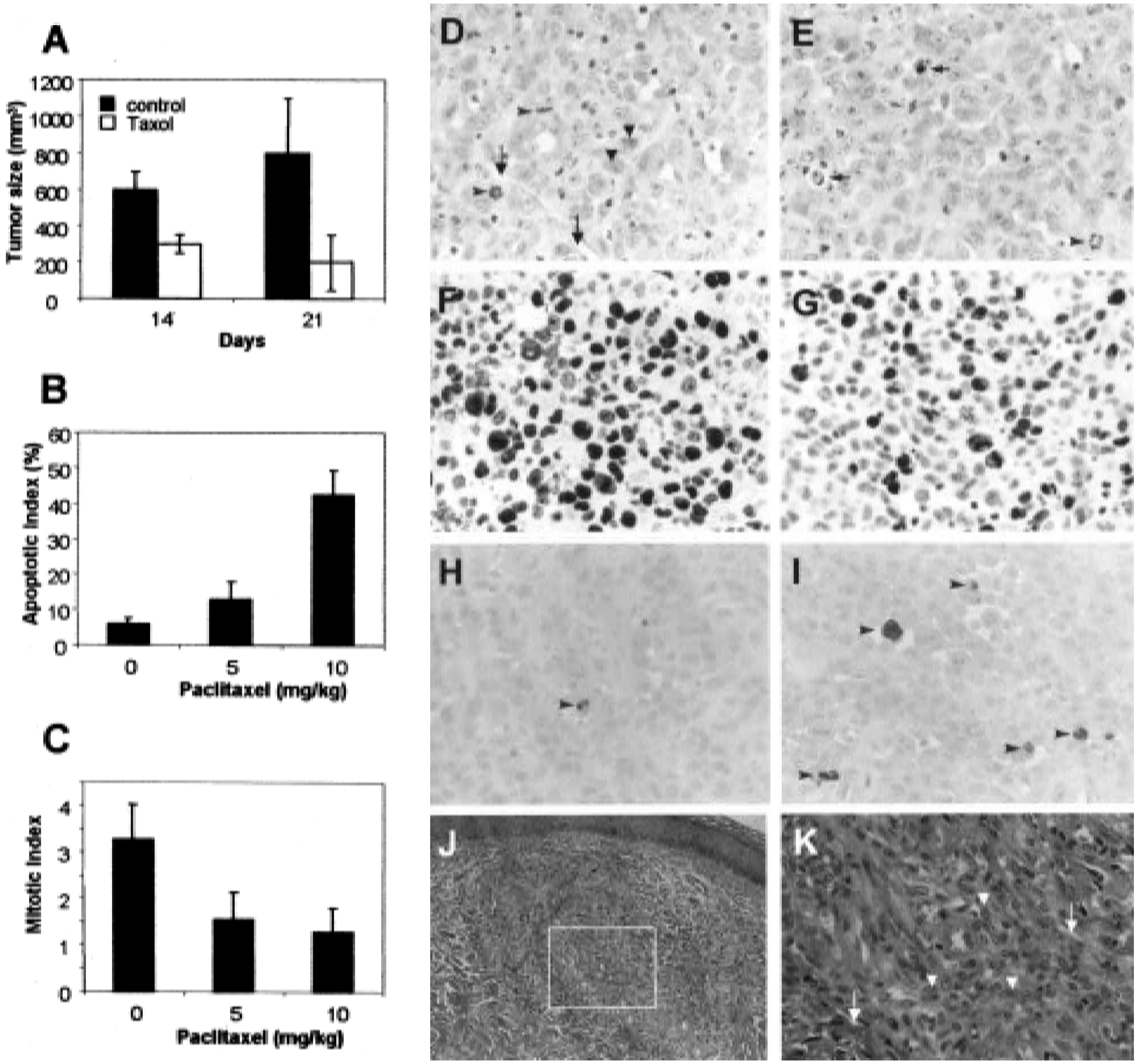

**Figure 6**. *Activity of paclitaxel on Kaposi's sarcoma tumor growth in mice. (a) Mice were treated either with paclitaxel or the control (6 mice per group) intraperitoneally (20 mg/kg) on days 1, 5, 9, 14 and 21. Tumors were measured on days 14 and 21. The results represent the mean ± standard error of tumor size. In other experiments, immunodeficient mice were injected with 10 million cells per mouse subcutaneously. Mice were treated with a single dose of paclitaxel at 5 or 10 mg intraperitoneally and the tumors were excised after 24 hours and examined for (b) apoptosis in situ and (c) mitotic index. Histology of taxol-treated tumors. H&E stained sections of control tumors (d) showed a cellular malignant tumor with frequent mitotic figures (horizontal arrow heads). (e) Taxol-treated tumors showed fewer mitotic figures (horizontal arrow heads) and scattered apoptotic bodies (horizontal arrows). (f) Immunoperoxidase staining for the proliferation related antigen Ki-67 using MIB-1 revealed a high labeling index in control tumors. (g) In taxol-treated tumors, the MIB-1 labeling index was significantly lower. (h) TUNEL staining for apoptotic cells revealed rare apoptotic cells (arrow heads) in control tumors. (i) Apoptotic cells were significantly more frequent in taxol treated cells (arrow heads). An H&E stained section of a typical KS lesion in an AIDS-KS patient is shown (j). The area that is magnified in (k) is indicated by the white rectangle. (k) High power view of KS biopsy for comparison to KS Y-1 tumor xenograft in mice shown in panels D-I. Both the KS Y-1 xenograft (d) and KS biopsy (k) show similar tumor cells (vertical arrowheads in both panels) and the occurrence of slit-like spaces typical of KS lesions (vertical arrows in both panels).*

nature we hypothesized that KS would be especially sensitive to paclitaxel, since both the apoptotic and anti-angiogenic properties of the drug would be in action against this tumor. We therefore set out to examine the response of KS cells *in vitro* and *in vivo* to paclitaxel.

KS cells were very sensitive to the cytotoxic effects of paclitaxel *in vitro*. The concentration of half-maximal growth inhibition (0.8 nM) was an order of magnitude lower than reported for several other tumor cell types.[40–42] The cytotoxic activity of paclitaxel in KS cells was due to apoptosis, and these effects could be seen at a dose as low as 0.1 nM *in vitro*. Two events indicative of apoptosis, characteristic DNA banding resulting from internucleosomal cleavage, and the appearance of annexin V on the outer surface of the cell membrane were both observed upon paclitaxel treatment of KS cells.

Having established that paclitaxel induces apoptosis in KS cells, we next undertook to examine the behavior of some of the important apoptotic molecules in these cells. We showed that two anti-apoptotic proteins, Bcl-2 and Bcl-$x_L$, were expressed in KS cells and phosphorylated after 24 hour treatment with paclitaxel. Phosphorylation of Bcl-2 has been regarded as a marker of apoptosis; however, recent results suggest that phosphorylated Bcl-2 is not in itself an apoptotic protein.[18,43] Bcl-2 phosphorylation occurs in tandem with mitosis as a normal part of the cell cycle and does not necessarily lead to apoptosis. Bcl-2 can be considered a cell cycle checkpoint protein that responds to mitotic spindle defects in analogy to the checkpoint protein p53 which responds to DNA damage. It is interesting that KS cells also express Bcl-$x_L$, which can compensate for Bcl-2 activity.[38] We noted that the levels of the pro-apoptotic protein Bax did not change in response to paclitaxel treatment. Presumably, the extended phosphorylation of both Bcl-2 and Bcl-$x_L$ in this system releases Bax from heterodimers with these molecules to exert its downstream apoptotic effects. Two of these documented effects are release of cytochrome c into the cytosol and activation of the caspase family of proteases. We demonstrated that cytochrome c was released at the concentrations that were used in the other *in vitro* assays. This is in agreement with results that demonstrate that Bcl-2 blocks the release of cytochrome c from the mitochondria and that Bax enables its release.[23,24] In turn cytosolic cytochrome c binds Apaf-1, activates caspase 9 and leads to activation of the caspases, including caspase 3, which are the effectors of apoptosis.[25,26] We thus showed activation of caspase 3 by cleavage of its pro form. In addition to showing that caspase-3 itself was cleaved we showed that it was indeed activated. Activation of caspase 3 coincided with the cleavage of caspase 3 substrates, poly (ADP-ribose) polymerase (PARP) and DNA fragmentation factor (DFF). In this KS cell system peak levels of phosphorylation of Bcl-2 and Bcl-$x_L$ preceded peak caspase-3 activity by at least 7 hours. This is in keeping with the current paradigm of apoptosis in which Bcl-2 phosphorylation leads to free Bax, which in turn leads to release of cytochrome c from the mitochondria, which then initiates the final phase of apoptosis where proteases are released to effect cell death.[23–26]

The *in vivo* results in KS tumor xenografts in nude mice mirror the *in vitro* results. There was significant (75%) reduction of tumor size in paclitaxel treated mice and histology of the tumors revealed a decrease in proliferation, a decrease in mitosis and extensive apoptosis of tumor cells compared to untreated controls. The major mechanism of paclitaxel anti-tumor activity in this model appears to be induction of apoptosis in the tumor cells. While we did show that *in vitro* paclitaxel inhibited KS cell migration, the contribution of anti-angiogenic effects of paclitaxel was not investigated *in vivo*. However, there is precedent for a multifaceted anti-angiogenic activity of paclitaxel. Not only does paclitaxel inhibit angiogenesis by interfering with migration of endothelial cells,[27] but also it induces macrophage production of the anti-angiogenic cytokine IL-12.[43] This last point may be of significance in KS, which is a tumor highly infiltrated with macrophages.

In conclusion, the anti-KS effect of paclitaxel can in large part be attributed to the apoptosis initiating activity of the drug; however, there is a component of anti-angiogenic action since we showed that KS cell migration was inhibited at low concentrations of paclitaxel.

### Acknowledgements

This study was supported in part by a research grant from Bristol–Myers Squibb, Pharmaceutical Research Institute and by the Bridges and Larson Foundation.

### References

1 Gill PS, Hamilton AW, Naidu Y. Epidemic (AIDS-related) Kaposi's sarcoma: epidemiology, pathogenesis, and treatment. *AIDS Updates* 1994;7:1–11.

2 Rabkin CS, Janz S, Lash A, Coleman AE, Musaba E, Liotta L, *et al.* Monocolonal origin of multicentric Kaposi's sarcoma Lesions. *N Engl J Med* 1997;336:988–93.

3 Gill PS, Tsai YC, Rao AP, Spruck CH 3rd, Zheng T, Harrington WA Jr., *et al.* Evidence for multiclonality in multicentric Kaposi's sarcoma. *Proc Natl Acad of Sci USA* 1998;95:8257–61.

4 Albini A, Aluigi MG, Benelli R, Berti E, Biberfeld P, Blasig C, *et al.* Oncogenesis in HIV-infection: KSHV and Kaposi's sarcoma. *Int J Oncol* 1996;9:5–8.

5 Chatlynne LG, Ablashi DV. Seroepidemiology of Kaposi's sarcoma-associated herpesvirus (KSHV). *Semin Cancer Biol* 1999;9:175–85.

6 Lunardi-Iskandar Y, Gill PS, Lam VH, Zeman RA, Michaels F, Mann DL, *et al.* Isolation and characterization of an immortal neoplastic cell line (KS Y-1) from AIDS-associated Kaposi's sarcoma. *J Natl Can Inst* 1995;87:974–81.

7 Fiorelli V, Gendelman R, Sirianni MC, Chang HK, Colombini S, Markham PD, *et al.* γ-interferon produced by CD8+ T cells infiltrating Kaposi's sarcoma induces spindle cells with angiogenic phenotype and synergy with HIV-1 Tat protein: an immune response to HHV-8 infection? *Blood* 1998;91:956–67.

8 Fiorelli V, Gendelman R, Samaniego F, Markham PD, Ensoli B. Cytokines from activated T cells induce normal endothelial cells to acquire the phenotypic and functional features of AIDS-Kaposi's sarcoma spindle cells. *J Clin Invest* 1995;95:1723–34.

9 Masood R, Cai J, Zheng T, Smith DL, Naidu Y, Gill PS. Vascular Endothelial Growth Factor/Vascular permeability factor (VEGF/VPF) in as autocrine growth factor for AIDS-KS. *Proc Natl Acad Sci USA* 1997;94:979–84.

10 Weninger W, Partanen TA, Breiteneder-Geleff S, Mayer C, Kowalski H, Mildner M, Pammer, *et al.* Expression of vascular endothelial growth factor receptor-3 and podoplanin suggests a lymphatic endothelial cell origin of Kaposi's sarcoma tumor cells. *Lab Invest* 1999;79:243–51.

11 McGarvey ME, Flore O, Masood R, Arora N, Zheng T, Smith DL, *et al.* Expression of endothelial cell specific receptor tyrosine kinases, Tie-1 and Tie-2, Flt-1, Flk-1 and Flt-4 in Kaposi's sarcoma cell lines and in primary tumor tissues. *Blood* 1998;92:538.

12 Ensoli B, Markham P, Kao V, Barillari G, Fiorelli V, Gendelman R, *et al.* Block of AIDS-Kaposi's sarcoma (KS) cell growth, angiogenesis and lesion formation in nude mice by antisense oligonucleotide targeting basic fibroblast growth factor: a novel strategy for the therapy of KS. *J Clin Invest* 1994;94:1736–46.

13 Rowinsky EK, Cazenave LA, Donehower RC. Taxol: a novel investigational antimicrotubule agent. *J Natl Cancer Inst* 1990;82:1247–59.

14 McGuire WP, Rowinsky EK, Rosenshein NB, Grumbine FC, Ettinger DS, Armstrong DK, *et al.* Taxol: a unique antineoplastic agent with significant activity in advanced ovarian epithelial neoplasms. *Ann Intern Med* 1989;111:273–9.

15 Holmes FA, Walters RS, Theriault RL, Forman AD, Newton LK, Raber MN, *et al.* Phase II trial of taxol, an active drug in metastatic breast cancer. *J Natl Cancer Inst* 1991;83:1779–1805.

16 Saville MW, Lietzau J, Pluda JM, Feuerstein I, Odom J, Wilson WH, *et al.* Treatment of HIV-associated Kaposi's sarcoma with paclitaxel. *Lancet* 1995;346:26–8.

17 Horwitz SB, Cohen D, Rao S, Ringel I, Shen H-J, Yang C-PH. Taxol: mechanisms of action and resitance. *Monographs, J Natl Cancer Inst* 1993;15:55–62.

18 Scatena CD, Stewart ZA, Mays D, Tang LJ, Keefer CJ, Leach SD, *et al.* Mitotic phosphorylation of Bcl-2 during normal cell cycle progression and taxol induced growth arrest. *J Biol Chem* 1998;273:30 777–84.

19 Reed JC. Regulation of apoptosis by bcl-2 family proteins and its role in cancer and chemoresistance. *Curr Opin Oncol* 1995;7:541–6.

20 Kroemer G. The proto-oncogene Bcl-2 and its role in regulating apoptosis. *Nature Medicine* 1997;3:614–20.

21 Oltvai ZN, Milliman CL, Korsmeyer SJ. Bcl-2 heterodimerizes in vivo with a conserved homolog, Bax, that accelerates programmed cell death. *Cell* 1993;74:609–19.

22 Gross A, Jockel J, Wei MC, Korsmeyer SJ. Enforced dimerization of BAX results in its translocation, mitochondrial dysfunction and apoptosis. *EMBO J* 1998;17:3878–85.

23 Yang J, Liu X, Bhalla K, Kim CN, Ibrado AM, Cai J, Peng TI, *et al.* Prevention of apoptosis by Bcl-2: release of cytochrome c from mitochondria blocked. *Science* 1997;275:1129–32.

24 Jurgensmeier JM, Xie Z, Deveraux Q, Ellerby L, Bredesen D, Reed JC. Bax directly induces release of cytochrome c from isolated mitochondria. *Proc Natl Acad Sci USA* 1998;95:4997–5002.

25 Li P, Nijhawan D, Budihardjo I, Srinivasula SM, Ahmad M, Alnemri ES, *et al.* Cytochrome c and dATP-dependent formation of Apaf-1/caspase-9 complex initiates an apoptotic protease cascade. *Cell* 1997;91:479–89.

26 Harvey KJ, Blomquist JF, Ucker DS. Commitment and effector phases of the physiological cell death pathway elucidated with respect to Bcl-2 caspase, and cyclin-dependent kinase activities. *Mol Cell Biol* 1998;18:2912–22.

27 Belotti D, Vergani V, Drudis T, Borsotti P, Pitelli MR, Viale G, *et al.* The microtubule-affecting drug paclitaxel has antiangiogenic activity. *Clin Cancer Res* 1996;2:1843–9.

28 Burt HM, Jackson JK, Bains SK, Liggins RT, Oktaba AM, Arsenault AL, *et al.* Controlled delivery of taxol from microspheres composed of a blend of ethylene–vinyl acetate copolymer and poly (d,l-lactic acid). *Cancer Lett* 1995;88:73–9.

29 Klauber N, Parangi S, Flynn E, Hamel E, Damato RJ. Inhibition of angiogenesis and breast cancer in mice by the microtubule inhibitors 2-methoxyestradiol and taxol. *Cancer Res* 1997;57:81–6.

30 Lunardi-Iskandar Y, Gill P, Lam VH, Zeman RA, Michaels F, Mann DL, *et al.* Isolation and characterization of an immortal neoplastic cell line (KS Y1) from AIDS-associated Kaposi's sarcoma. *J Natl Cancer Inst* 1995;87:974–81.

31 Siegal B, Levinton-Kriss S, Schiffer A, Sayar J, Engelberg I, Vonsover A, *et al.* Kaposi's sarcoma in immunosuppression. Possibly the results of dual viral infection. *Cancer* 1990;65:492–8.

32 Ray S, Ponnathpur V, Huang Y, Tang C, Mahoney ME, Ibrado AM, *et al.* 1-β-D-arabinofuranosylcytosine-, mitoxantrone- and paclitaxel-induced apoptosis in HL-60 cells: improved method for detection of internucleosomal DNA fragmentation. *Cancer Chemother Pharmacol* 1994;34:365–71.

33 Darzynkiewicz Z, Bruno S, Del Bino G, Gorczyca W, Hotz MA, Lassotta P, *et al.* Features of apoptotic cells measured by flow cytometry. *Cytometry* 1992;13:795–808.

34 Bhalla K, Ibrado AM, Tourkina E, Tang CQ, Mahoney ME, Huang Y. Taxol induces internucleosomal DNA fragmentation associated with programmed cell death in human myeloid leukemic cell. *Leukemia* 1993;7:563–8.

35 Ibrado AM, Huang Y, Fang G, Bhalla K. Bcl-$x_L$ overexpression inhibits Taxol-induced Yama protease activity and apoptosis. *Cell Growth and Differentiation* 1996;7:1087–94.

36 Jordan MA, Wendell K, Gardiner S, Derry WB, Copp H, Wilson L. Mitotic block induced in HeLa cells by low concentrations of paclitaxel (Taxol) results in abnormal mitotic exit and apoptotic cell death. *Cancer Res* 1996;56:816–25.

37 Martin SJ, Green DR. Protease activation during apoptosis: death by a thousand cuts? *Cell* 1995;82:349–52.

38 Ibrado AM, Liu L, Bhalla K. Bcl-$x_L$ overexpression inhibits progression of molecular events leading to paclitaxel-induced apoptosis of human AML HL-60 cells. *Cancer Res* 1997;47:1109–15.

39 Gill PS, Tulpule A, Espina BM, Cabriales S, Bresnahan J, Ilaw M, *et al.* Paclitaxel is safe and effective in the treatment of AIDS-related Kaposi's sarcoma. *J Clin Oncol* 1999;17:1876–83.

40 Tortora G, di Isernia G, Sandomenico C, Bianco R,

Pomatico G, Pepe S, *et al*. Synergistic inhibition of growth and induction of apoptosis by 8-chloro- cAMP and paclitaxel or cisplatin in human cancer cells *Cancer Res* 1997;57:5107–11.

41 Roth W, Wagenknecht B, Grimmel G, Dichgans J, Weller M. Taxol-mediated augmentation of CD95 ligand-induced apoptosis of human malignant glioma cells: association with bcl-2 phosphorylation but neither activation of p53 nor $G_2$M cell cycle arrest. *Br J Cancer* 1998;77:404–11.

42 Gagandeep S, Novikoff PM, Ott M, Gupta S. Paclitaxel shows cytotoxic activity in human hepatocellular carcinoma cell lines. *Cancer Lett* 1999;136:109–18.

43 Mullins DW, Burger CJ, Elgert KD. Paclitaxel enhances macrophage IL-12 production in tumor-bearing hosts through nitric oxide. *J Immunol* 1999;162:6811–8.
